# Relationship between Entrepreneurship Education and Entrepreneurial Intention among College Students: A Meta-Analysis

**DOI:** 10.3390/ijerph191912158

**Published:** 2022-09-26

**Authors:** Wenyi Zhang, Yixing Li, Qing Zeng, Minqiang Zhang, Xiaozhong Lu

**Affiliations:** 1 School of Business Administration, Guangdong University of Finance and Economics, Guangzhou 510320, China; 2 School of Education, South China Normal University, Guangzhou 510631, China; 3 School of Psychology, South China Normal University, Guangzhou 510631, China; 4 Key Laboratory of Brain, Cognition and Education Sciences, Ministry of Education, South China Normal University, Guangzhou 510631, China; 5 School of Psychology, Center for Studies of Psychological Application, South China Normal University, Guangzhou 510631, China; 6 Guangdong Key Laboratory of Mental Health and Cognitive Science, South China Normal University, Guangzhou 510631, China

**Keywords:** entrepreneurship education, entrepreneurial intention, meta-analysis, moderating effect

## Abstract

Meta-analysis was used to investigate the impact of entrepreneurship education on college students’ entrepreneurial intention. Based on the rules of meta-analysis, 389 empirical studies were selected from more than 1000 entrepreneurship education-related documents. The current study processed and analyzed data from 36 records (including 24 journal articles, 11 master’s theses, and 1 doctoral thesis) with a total sample of 29,736 students. The results suggested that: (1) entrepreneurship education is positively associated with entrepreneurial intention; (2) national context differences among students have a significant and moderating effect on the relationship between entrepreneurship education and entrepreneurial intention. As compared to other countries, Chinese college students’ entrepreneurial intention is more strongly related with entrepreneurship education.

## 1. Introduction

With the globalization of the world economy and the normalization of the state of affairs after the COVID-19 epidemic, college students’ entrepreneurship is characterized by new opportunities and challenges [1]. According to the data of the “2019 Chinese College Students Employment Report” released in July 2020, the proportion of self-employed undergraduates (four-year course) in 2019 was 1.6%, while that of the technical graduates (three-year course) was 3.4%. A previous study has shown that the proportion of graduates who start their own businesses was around 8.1% within three years after graduation, but the proportion of self-employment is relatively low [2]. CEOWORLD magazine has assessed the 100 economies which collectively account for 95 percent of the global GDP, and has developed the “Best Countries to Start a Business” index by considering factors such as innovation, competitiveness, infrastructure, workforce skills, capital access, and openness of businesses. This magazine reported that China’s 2021 entrepreneurial activity index was 20.04, while the US and Germany’s entrepreneurial activity index were as high as 41 or more, indicating that China still lags behind in entrepreneurial activities as compared to the Western countries [3]. Entrepreneurship is a high-risk economic activity; its creativity, ambiguity, and limited resources are challenges for entrepreneurs. In particular, for college students who lack social experience and connections, the uncertainty and ambiguity is amplified [4]. Additionally, college students are usually lacking in mental preparedness for the hardship of entrepreneurial activities and in entrepreneurial skills, while the social environment for young entrepreneurs is normally not as friendly as that for experienced and older entrepreneurs [5]. Therefore, it is necessary to further investigate the entrepreneurial intention of college students, and to explore the relationship between entrepreneurship education and entrepreneurial intention. From a practical point of view, the current study enables policy makers and educators to obtain targeted advice on building an entrepreneurship education system that integrates professional and entrepreneurship education.

## 2. Literature Review

### 2.1. Entrepreneurship Education and Entrepreneurial Intention

Entrepreneurship education refers to the educational curriculum and courses that aim to cultivate entrepreneurial spirits and competencies in students, such as identifying opportunities, integrating resources, and developing ventures [6]. In 1946, entrepreneurship education was first introduced in the entrepreneurship course at Harvard Business School. In 1967, Stanford University and New York University introduced entrepreneurship courses in their MBA programs [7]. Entrepreneurship education not only helps to promote students’ entrepreneurial intention, but also helps develop an entrepreneurial way of thinking and cultivates skills; additionally, it plays an important role in promoting the growth of human capital [8]. Furthermore, entrepreneurship education is the driving force that promotes the development of entrepreneurial ability [9], which helps in improving individual’s entrepreneurial competitiveness [10]. Entrepreneurial intention is the subjective state of mind of potential entrepreneurs that determines whether to engage in entrepreneurial activities; it is also the willingness of individuals towards entrepreneurial behaviors such as starting a new business or becoming an entrepreneur [11]. Human Capital Theory (HCT) indicates that entrepreneurial intention is determined on the basis of the human and social capital stock [12]. The growth of entrepreneurial professional knowledge and skills accumulated through entrepreneurship education can improve an individual’s entrepreneurial intention.

According to the Theory of Planned Behavior (TPB), an individual’s behavior is influenced by personal attitude, subjective norm, and perceptual behavior control [13]. Personal attitude refers to an individual’s evaluation of a target, and individuals with positive evaluation are usually willing to carry out such behavior [13]. Subjective norm is the social pressure that individuals experience when they perform a certain behavior. Perceptual behavior control has been defined as an individual’s subjective judgments about whether they can complete expected goals successfully [13]. Entrepreneurship education not only promotes college students to learn the methods and skills needed to start a business, but also relates to students’ positive evaluation of entrepreneurship and to the belief in their ability to complete the goal successfully [13]. More importantly, entrepreneurship education improves the entrepreneurial intention of college students. Previous studies have found that entrepreneurship education is positively related to entrepreneurial intention. Entrepreneurship education was found to improve entrepreneurial intention among college students from Tianjin and Qinhuangdao in China [14]. Entrepreneurship education is positively associated with college students’ entrepreneurial intention [15]. Furthermore, entrepreneurship education plays a key role in college students’ entrepreneurial intention by providing awareness, knowledge, and ability [16]. However, some studies suggest that entrepreneurship education is not significantly related to entrepreneurial intention [17,18]. Thus, the current study aims to investigate the relationship between entrepreneurship education and entrepreneurial intention among college students.

**Hypothesis** **1:** *There is a positive correlation between college students’ entrepreneurship education and entrepreneurial intention.*

### 2.2. Moderating Role of National Context Differences

National context differences play an important role in the relationship between entrepreneurship education and entrepreneurial intention. Entrepreneurship education is considered highly important and has developed rapidly in the governments and universities of the United States and Europe [19,20]. To facilitate further development of entrepreneurship education, governments and schools offer entrepreneurship courses and organize extracurricular activities such as entrepreneurship competitions [21]. For example, in Europe, a competition pertaining to entrepreneurship teaching methods is held in schools, in which teacher groups participate and entrepreneurs are invited as judges [22]. Another important way to promote the development of entrepreneurship education is organizing Cross-Campus Entrepreneurship Education (CCEE) groups, in which business schools play a core role and multi-department cooperation is required. Supported by their sound social and economic foundation and policy, some countries in Europe and the United States have formed a well-rounded educational system with entrepreneurship education as the main body and have sought interdisciplinary integration [23]. However, entrepreneurship education in China started relatively late, and was mostly restricted to classrooms only, with less practical application [24]. In China, the concept of innovation and entrepreneurship education was first introduced in 2010, and at the same time the “Entrepreneurship Education Steering Committee of Higher Education Institutions” was established to promote professional development through entrepreneurship education [25]. In 2012, China promulgated the basic syllabus for entrepreneurship education, which carefully outlined the entrepreneurship teaching curriculum system for undergraduate schools [26]. In 2015, China began to build entrepreneurship incubation and demonstration bases, hold competitions pertaining to innovation and entrepreneurship for college students, facilitate teachers’ innovation and the development of entrepreneurship teaching capabilities, and establish an effective linkage between universities, enterprises, and society [27]. Currently, with the increasing popularization of higher education, it has been reported that there were 10.76 million college graduates in China in 2022, which shows an increase of 1.67 million compared with those in 2021 [28]. As a driving force of the economy, entrepreneurship education can provide job opportunities and play a significant role in promoting employment [15]. Therefore, it is necessary to pay more attention to how national context differences affect the relationship between entrepreneurship education and college students’ entrepreneurial intention.

**Hypothesis** **2:** *National context differences can significantly affect the relationship between entrepreneurship education and entrepreneurial intention.*

### 2.3. Moderating Roles of Gender

Gender and education level are important factors that influence the relationship between entrepreneurship education and college students’ entrepreneurial intention. While preparing to start a business, females face greater entrepreneurial pressure than males; they are often bound by sexist and traditional beliefs, such as the belief that males should work outside and females should stay at home [29]. Some scholars attribute this to different perceptions of the social roles of males and females during childhood, leading to role consciousness in men, which is characterized by independence and a focus on work, while females tend to pay more attention to interpersonal relationships and also tend to prioritize family needs over occupational needs [29]. Furthermore, prior research has indicated that males’ entrepreneurial intention is positively related to their entrepreneurship education, while females are less affected by entrepreneurship education [30]. Therefore, it can be assumed that gender plays a moderating role in the relationship between entrepreneurship education and entrepreneurial intention.

**Hypothesis** **3:** *Gender plays a moderating role between entrepreneurship education and entrepreneurial intention.*

### 2.4. Education Level

In addition, there is a traditional view that individuals with higher educational qualifications have lower entrepreneurial intention. With the improvement in educational level, students with longer educational experience tend to work in a stable and better-paid state-owned enterprise, and avoid entrepreneurial risks [31]. Another study has suggested that the low proportion of postgraduate entrepreneurs should be attributed to the fact that the collaborative system of entrepreneurship education has not been established [32]. Graduate students have a positive attitude towards entrepreneurial behavior, but their own entrepreneurial intention is not strong [33]. On the one hand, the educational level of an individual is closely related to factors such as personal comprehensive quality and their social network, which serve as key resources for entrepreneurship; on the other hand, the consideration of reputation and risk factors may inhibit the entrepreneurial intention of highly educated individuals [34]. Therefore, this study aimed to investigate the impact of education level on entrepreneurial intention.

**Hypothesis** **4:** *Education level plays a moderating role between entrepreneurship education and entrepreneurial intention.*

## 3. Materials and Methods

### 3.1. Literature Search

The data for meta-analysis were collected from the literature published from 2010 to 2020. The current study selected JSTOR, Springer, Google Scholar, CNKI, and Wanfang Data Knowledge Service Platform as the databases to search the literature related to entrepreneurship education and entrepreneurial intention. With the outbreak of COVID-19, the academics and employment of college students were negatively affected [35,36,37]. Hence, studies conducted in 2021 were not included in the meta-analysis. 

### 3.2. Literature Inclusion and Exclusion

To meet the requirements of meta-analysis methods and the aim of this work, 389 empirical studies were selected from more than 1000 entrepreneurship education-related documents, which were further filtered according to the following criteria: (a) the research sample should cover college students, including technical college students (three-year higher education system), undergraduate students (four-year higher education system), and graduate students; (b) the literature must be empirical, excluding literature reviews and theoretical articles; (c) the literature must report a correlation to express the relationship between entrepreneurship education and entrepreneurial intention, in the form of a correlation coefficient; (d) only one of the multiple studies on the same samples should be included; (e) the sample size is clear. Hence, after searching and filtering, 36 studies were finally obtained (see Table 1), including journal articles (24), master’s theses (11), and a doctoral thesis (1).

### 3.3. Data Analysis

Meta-analysis highlights the differences between specific studies, comprehensively evaluates related studies, and reveals conclusions or trends at a more macroscopic level that have greater value [38]. Data processing is based on the rules of meta-analysis. The effect size was based on the sample of each independent study. The current study coded the relationship between college students’ entrepreneurial education and entrepreneurial intention as follows: (a) if only one correlation coefficient was reported in the study, then the correlation coefficient was regarded as an independent effect value; (b) if the correlation coefficients reported in the study were from different groups, then each correlation coefficient was regarded as an independent correlation effect value, and was included in the analysis; (c) if the coefficients expressed the relationship between different dimensions of entrepreneurship education and entrepreneurial intention in the same group, then the mean of these correlation coefficients was calculated and expressed as the effect value. 

The CMA 3.0 software was used in this study. The correlation coefficient was used as the input effect value. Firstly, each r-value was converted to a Fisher’s Z value. Secondly, the weighted average of the Fisher’s Z values was calculated. Finally, the Fisher’s Z values were converted back to the correlation coefficient to obtain the final effect value. The effect values of each sample are depicted in Table 1.

**Table 1:** Meta-analysis literature summary.

First Author	Publication Year	Literature Type	Sample Size	Country	Gender	Whether the Sample Includes Graduate Students	Effect Size
Wang [39]	2010	Journal	646	China	Female	No	0.244
Dohse [40]	2010	Journal	1969	Germany	Male	No	0.055
Keat [41]	2011	Journal	417	Malaysia	Female	No	0.319
Lanero [42]	2011	Journal	800	Spain	Female	No	0.15
Wang [14]	2012	Journal	143	China	Female	No	0.314
Liu [43]	2013	Journal	339	China	Uncertain	No	0.438
Karali [44]	2013	Thesis	13,121	Netherlands	Female	Yes	0.0444
Solesvik [45]	2013	Journal	189	Ukraine	Female	Yes	0.21
Tang [46]	2014	Thesis	206	China	Uncertain	No	0.198
Shinnar [47]	2014	Journal	187	America	Male	No	0.3825
Yan [48]	2015	Thesis	370	China	Male	No	0.46
Shi [49]	2015	Journal	215	China	Male	No	0.696
Tan [50]	2015	Journal	305	China	Female	No	0.449
Du(a) [51]	2015	Thesis	208	China	Male	No	0.704
Du(b) [51]	2015	Thesis	176	Germany	Male	No	0.519
Fayolle [52]	2015	Journal	158	America	Uncertain	No	0.41
Mustapha [53]	2015	Journal	178	Malaysia	Female	No	0.426
Piperopoulos [54]	2015	Journal	114	Britain	Male	No	0.21
Huang [55]	2016	Journal	231	China	Male	No	0.146
Yan [56]	2016	Thesis	310	China	Female	No	0.22
Zhang [57]	2017	Thesis	354	China	Female	Yes	0.379
Kong [58]	2017	Journal	698	China	Male	No	0.421
Gao [59]	2018	Thesis	383	China	Male	Yes	0.459
Nabi [60]	2018	Journal	150	Britain	Female	No	0.303
Zhang [61]	2019	Thesis	450	China	Female	Yes	0.409
Xu [2]	2019	Journal	403	China	Female	No	0.631
Tao [62]	2019	Journal	336	China	Female	No	0.772
Sun [63]	2019	Journal	736	China	Female	No	0.21
Yu [64]	2019	Journal	333	China	Male	Yes	0.646
Xu [65]	2020	Journal	454	China	Male	No	0.357
Chen [66]	2020	Thesis	453	China	Female	No	0.188
Li [67]	2020	Thesis	3341	China	Uncertain	No	0.571
Wang [68]	2020	Journal	432	China	Female	No	0.443
Yu [69]	2020	Journal	497	China	Uncertain	No	0.365
Wang [70]	2020	Thesis	336	China	Male	Yes	0.487
Hua [71]	2020	Journal	98	China	Uncertain	No	0.3328

## 4. Results

### 4.1. The Main Effect Size of Entrepreneurship Education and Entrepreneurial Intention

#### 4.1.1. Heterogeneity Test

The heterogeneity test aims to examine whether the samples in each article belonged to the same group. There are two indexes for testing heterogeneity in meta-analysis, namely the Q-value and I2. When the result of the chi-square test with respect to the Q-value is significant (*p* < 0.05), it indicates that there is heterogeneity among the samples; the value of I2 lies between 0 and 100%, where 25%, 50%, and 75% represent low, medium, and high heterogeneity, respectively. When the heterogeneity is significant, a random effects model is used for analysis; otherwise, a fixed effect model is used. In this study, with respect to the results of the meta-analysis, Q (35) = 1951.970, *p* < 0.001, I2 = 98.207. This shows that 98.207% of the total variance is caused by the difference in the relationship between the college students’ entrepreneurship education and entrepreneurial intention. The results of the heterogeneity test indicated that a random effects model should be selected for the follow-up analysis.

#### 4.1.2. Publication Bias Test

Publication bias refers to the phenomenon that statistically significant findings are more likely to be published than non-significant findings. That is, only those studies that have significant results are published in journals, while those having non-significant results are excluded. Thus, publication bias is likely to affect the results of the meta-analysis. Therefore, the meta-analysis effect size must be tested before the meta-analysis to avoid the interference of publication bias [72,73]. In this study, the fail-safe N was used as an indicator of publication bias. If the fail-safe N value is greater than the tolerance value (5K + 10, where K represents the number of independent samples included in the study), it indicates that the meta-analysis results are not affected by publication bias. In this study, the fail-safe N value was 2627, which was greater than the tolerance value of 190, which indicated that the influence of publication bias in this study could be considered insignificant and could be ignored.

#### 4.1.3. Main Effect Size Test 

As shown in Table 2, the main effect of entrepreneurship education and the entrepreneurial intention of college students reveals a significant correlation between the two (r = 0.394, *p* < 0.001), and its confidence interval was [0.310, 0.472]. This shows that there is a positive correlation between college students’ entrepreneurship education and entrepreneurial intention, and thus Hypothesis 1 was accepted.

### 4.2. Analysis of Moderating Effects 

This study attempted to explain the heterogeneity of the relationship between entrepreneurship education and entrepreneurial intention by considering the role of the moderating factors (i.e., national context differences, gender, and education level). The results in Table 3 show that national context differences can significantly affect the relationship between entrepreneurship education and entrepreneurial intention (Qb = 8.920, *p* = 0.003); in China, there is a positive relationship between them (r = 0.440), while in other countries, the relationship is weaker as compared to China (r = 0.271), so Hypothesis 2 was also accepted.

The test results pertaining to the moderating role of gender show that the influence of male groups on entrepreneurship education and entrepreneurial intention (r = 0.446) is stronger than that of female groups (r = 0.352), but the difference between the two groups was non-significant (*p* = 0.558). Therefore, gender does not play a moderating role between entrepreneurship education and entrepreneurial intention, and hence Hypothesis 3 was rejected.

The results of the moderating effect test on education level have shown that the effect size of the group of non-graduate students (r = 0.403) was stronger than that of the group of graduate students (r = 0.362), but the heterogeneity between the groups was not significant (*p* = 0.691). This indicated that the moderating effect of education level was not significant, and hence Hypothesis 4 was also rejected.

## 5. Discussion

Meta-analysis is a statistical reanalysis of previous research results based on specific conditions and topics [37]. Compared with ordinary empirical research, the advantage of meta-analysis is that it breaks through contingencies and limitations, and overcomes the problem of result distortion caused by errors [74]. Considering the advantages of meta-analysis, the current study attempts to explore the literature to analyze the relationship between entrepreneurship education and entrepreneurial intention from a global perspective. Additionally, prior research has suggested that entrepreneurship education has an interactional effect with other variables, such as gender, in regard to its relationship with entrepreneurial behavior and entrepreneurial intention [29]. Thus, it is necessary to sort out the literature in the field of entrepreneurship education, and to further explore the relationship between entrepreneurship education and entrepreneurial intention. This research used the method of meta-analysis to statistically analyze 36 studies pertaining to the relationship between entrepreneurship education and entrepreneurial intention published in the past ten years. The moderating effect of national context differences, gender, and education level on the relationship between entrepreneurship education and entrepreneurial intention was discussed, and the following conclusions were drawn.

### 5.1. Effect of Entrepreneurship Education on Entrepreneurial Intention

The main effect of entrepreneurship education on the entrepreneurial intention of college students shows that the random effect size of the correlation coefficient between entrepreneurship education and entrepreneurial intention was 0.394, which reached a significant level, indicating that entrepreneurship education has a positive role in promoting entrepreneurial intention. This is consistent with the findings of previous studies [14,16] which revealed that entrepreneurship education is positively associated with the entrepreneurial intention of college students. Therefore, entrepreneurship education is essential to improve the entrepreneurial intention of college students. Universities should develop and implement an entrepreneurship education curriculum system, which includes compulsory courses, elective courses, and practical courses, among others. Additionally, entrepreneurship education should be provided throughout the learning period at the college level. Governments and schools can build an entrepreneurship education system that integrates professional and entrepreneurship education. Furthermore, they can encourage students to participate in innovation and entrepreneurship competitions and practices. By encouraging high-quality projects on campus, providing space and entrepreneurial incubators, and implementing other practical methods, they can increase the frequency of college students’ entrepreneurial behavior. At the same time, this is likely to enhance entrepreneurial intention and increase entrepreneurial confidence.

### 5.2. Role of National Context Differences

National context differences have a moderating effect on the relationship between entrepreneurship education and entrepreneurial intention, and the positive relationship between the two is stronger in China than that in other countries. Hypothesis 2 is established. The results of the study show that as compared to the United States, the United Kingdom, Germany, Malaysia and other countries, the entrepreneurial intention of Chinese college students is more affected by entrepreneurship education. An important indicator in the world-renowned international entrepreneurship research project, the Global Entrepreneurship Observatory, is total early-stage entrepreneurial activity (TEA), which mainly measures two dimensions: individual entrepreneurial behavior and social surroundings. In the Global Entrepreneurship Watch 2019/2020 report, the 18- to 24-year-old population in China accounted for 10.6% of the overall measured population with early-stage entrepreneurial behavior, as compared to 15.8% in the United States and 12.2% in the United Kingdom. It can be seen that although the proportion of early-stage entrepreneurial behavior among people aged 18–24 years in China has shown an upward trend in recent years, it still lags behind other countries such as the United Kingdom and the United States. One possible explanation is that countries such as the United Kingdom and the United States have a strong entrepreneurial culture; their college students exhibit entrepreneurial behavior irrespective of whether they receive entrepreneurial education or not. Countries such as the United Kingdom and the United States have mature systems and policy support for financing college students’ entrepreneurial endeavors; in particular, the entrepreneurial environment for young people is loose. These factors may be related to the results presented in the Global Entrepreneurship Watch. The entrepreneurial intention of Chinese college students is more affected by their entrepreneurship education. 

### 5.3. Role of Gender

The results of the meta-analysis revealed that there is a slight moderating effect of gender differences on the relationship between entrepreneurship education and entrepreneurial intention, but this effect is not significant. This shows that, as compared to males, the entrepreneurial intention of females is less affected by entrepreneurship education. After examining the entrepreneurship education courses, it can be concluded that entrepreneurship education is gender neutral. When males and females receive the same entrepreneurship education, the impact on their entrepreneurial intention is basically the same. However, we still need to pay attention to the differential needs of females. Entrepreneurship can increase employment opportunities, the social status of females, and help them realize their self-worth. At the same time, the active participation of females in entrepreneurship is also in line with the needs of social and economic development [75]. Therefore, entrepreneurship education in universities can appropriately enrich the content related to female entrepreneurship. In the design of entrepreneurship courses and for the realization of entrepreneurship projects, more attention should be paid to the needs of female entrepreneurs in order to understand the difficulties and bottlenecks they encounter in entrepreneurship, and to overcome the barriers to entrepreneurship.

### 5.4. Role of Education Level

Additionally, the moderating effect of education level in the relationship between entrepreneurial intention and entrepreneurship education is different. This is consistent with the conclusion that highly educated people are more inclined to have stable employment rather than take entrepreneurial risks. However, with the gradual popularization and development of entrepreneurship education in the field of higher education, entrepreneurship in postgraduates is also expected to rise. To stimulate the entrepreneurial passion of highly educated talents, cultivate their entrepreneurial willingness, and establish an entrepreneurial education system, the psychological needs of the students should be considered in future entrepreneurship education.

## 6. Conclusions and Future Direction

The entrepreneurship education of college students has a positive role in promoting entrepreneurial intention. National context differences have a significant moderating effect on the relationship between entrepreneurship education and entrepreneurial intention. As compared to college students from other countries, Chinese college students’ entrepreneurial intention is more strongly affected by entrepreneurship education. One of the purposes for college students to receive higher education is to improve the students’ employment competitiveness. Entrepreneurship education brings professional knowledge and skills to students, and some students will start their own businesses after graduating from university. Obviously, in China, entrepreneurship education plays a positive role in promoting college students’ entrepreneurial intention. Therefore, it is necessary to better organize and construct the curriculum system of entrepreneurship education in college. However, there are still several limitations to this study, and further research should be conducted. First, based on the literature review and theoretical support, this study investigated the impact of entrepreneurship education on college students’ entrepreneurial intention through meta-analysis. The moderating effect of national context differences, gender, and education level on the relationship between entrepreneurship education and entrepreneurial intention was also discussed. However, the influence of other factors, such as personality traits and entrepreneurial self-efficacy, on entrepreneurship education and entrepreneurial intention needs to be further explored. Second, all the studies selected in this work were cross-sectional. Future studies can choose longitudinal studies to explore the causal relationship between entrepreneurship education and entrepreneurial intention.

## Figures and Tables

**Table 2:** Meta-analysis results of sample main effects.

Model	Number of Studies	Effect Size and 95% Confidence Interval			Two-Sided Test	
		Estimate	Lower Interval	Upper Interval	*z*-Value	*p*-Value
Fixed effects	36	0.247	0.237	0.258	43.466	0.000
Random effects	36	0.394	0.310	0.472	8.499	0.000

**Table 3:** Meta-analysis results of moderation effect analysis.

Moderator		Number of Studies	Effect Size	95% Confidence		Two-Sided Test		Between-Group Heterogeneity		
				Lower Interval	Upper Interval	z-Value	p-Value	$Q_{b}$-Value	$df\left(Q\right)$	p-Value
Country	China	25	0.440	0.369	0.507	10.820	0.000	8.920	1	0.003
	Other countries	11	0.271	0.181	0.356	5.753	0.000			
Gender	Male	13	0.446	0.310	0.564	5.921	0.000	1.166	2	0.558
	Female	17	0.352	0.236	0.459	5.648	0.000			
	Uncertain	6	0.397	0.258	0.520	5.274	0.000			
Educational qualification	Including graduate students	8	0.362	0.162	0.533	3.450	0.000	0.158	1	0.691
	Does not include graduate students	28	0.403	0.319	0.481	8.649	0.000			

## Data Availability

The data are not publicly available due to privacy restrictions.
